# Bright light in elderly subjects with nonseasonal major depressive disorder: a double blind randomised clinical trial using early morning bright blue light comparing dim red light treatment

**DOI:** 10.1186/1745-6215-9-48

**Published:** 2008-07-31

**Authors:** Ritsaert Lieverse, Marjan MA Nielen, Dick J Veltman, Bernard MJ Uitdehaag, Eus JW van Someren, Jan H Smit, Witte JG Hoogendijk

**Affiliations:** 1 Department of Psychiatry, VU University Medical Center and Academic Outpatient Clinic for Affective Disorders, Stichting GGZBuitenamstel-de Geestgronden, AJ Ernststraat 887, 1081HL, Amsterdam, The Netherlands; 2 Center for Neurosciences Neurogenomics and Cognitive Research (CNCR), VU University Medical Center, De Boelelaan 1085, 1081 HV, Amsterdam, The Netherlands; 3 Netherlands Institute for Neuroscience, Royal Netherlands Academy of Arts and Sciences, Meibergdreef 47, 1105 BA, Amsterdam, The Netherlands; 4 Clinical Epidemiology and Biostatistics, VU University Medical Center, Amsterdam, The Netherlands

## Abstract

**Background:**

Depression frequently occurs in the elderly. Its cause is largely unknown, but several studies point to disturbances of biological rhythmicity. In both normal aging, and depression, the functioning of the suprachiasmatic nucleus (SCN) is impaired, as evidenced by an increased prevalence of day-night rhythm perturbations, such as sleeping disorders. Moreover, the inhibitory SCN neurons on the hypothalamus-pituitary adrenocortical axis (HPA-axis) have decreased activity and HPA-activity is enhanced, when compared to non-depressed elderly. Using bright light therapy (BLT) the SCN can be stimulated. In addition, the beneficial effects of BLT on seasonal depression are well accepted. BLT is a potentially safe, nonexpensive and well accepted treatment option. But the current literature on BLT for depression is inconclusive.

**Methods/Design:**

This study aims to show whether BLT can reduce non-seasonal major depression in elderly patients. Randomized double blind placebo controlled trial in 126 subjects of 60 years and older with a diagnosis of major depressive disorder (MDD, DSM-IV/SCID-I). Subjects are recruited through referrals of psychiatric outpatient clinics and from case finding from databases of general practitioners and old-people homes in the Amsterdam region. After inclusion subjects are randomly allocated to the active (bright blue light) vs. placebo (dim red light) condition using two Philips Bright Light Energy boxes type HF 3304 per subject, from which the light bulbs have been covered with bright blue- or dim red light- permitting filters. Patients will be stratified by use of antidepressants. Prior to treatment a one-week period without light treatment will be used. At three time points several endocrinological, psychophysiological, psychometrically, neuropsychological measures are performed: just before the start of light therapy, after completion of three weeks therapy period, and three weeks thereafter.

**Discussion:**

If BLT reduces nonseasonal depression in elderly patients, then additional lightning may easily be implemented in the homes of patients to serve as add-on treatment to antidepressants or as a stand-alone treatment in elderly depressed patients. In addition, if our data support the role of a dysfunctional biological clock in depressed elderly subjects, such a finding may guide further development of novel chronobiological oriented treatment strategies.

**Trial registration:**

ClinicalTrials.gov identifier: NCT00332670

## Background

Depression frequently occurs in the elderly. Prevalence figures of late-life depression of clinical relevance vary between 9% and 18% [1]. The effects of depressive disorders on well-being, self-sufficiency and daily functioning are enormous and and comparable to those of major chronic physical illnesses, and have clear economic consequences [1-6].

The cause of depression is unknown. However, the circadian system and a decreased activity of the suprachiasmatic nucleus (SCN), which is known as the 'biological clock' seems to be involved [7]. The age-dependent decrease of SCN activity could therefore be a risk factor for aged people to develop circadian rhythm-dependent perturbations and HPA-axis hyperactivity and depressive symptoms [8]. Disruptions in biological rhythms are known to be strongly associated with mood disorders. Indeed some of the major hallmarks of major depressive disorder (MDD) are abnormal sleep/wake, appetite, and social rhythms [9-11]. Depression symptoms are also diurnal with the most severe symptoms occurring typically in the morning [12], and depression is more prevalent in areas of the world that receive little sunlight for extended periods of time [13]. In addition, nearly all of the successful treatments for mood disorders seem to affect circadian rhythms, and it appears that the shifts, resetting and stabilization of these rhythms produced by these treatments are important for therapeutic efficacy.

### Advantages of light therapy

Patients accept and often prefer non-pharmacological treatments. The few systematic reports of side-effects suggest it is safe with only few counter indications. Light therapy also provides a viable alternative for patients who refuse, resist or cannot tolerate medication.

### Bright light therapy in Seasonal Major Depression

Bright light therapy (BLT) is considered a well and effective treatment of first choice for Seasonal Affective Disorder (SAD) [14], with an onset of action after only few days of treatment [15], and with a low side effect profile [16]. Its efficacy in SAD is comparable to that of antidepressant medication [17].

### Bright light therapy in Nonseasonal Major Depression

Trials on light therapy in nonseasonal depression have been overviewed by Kripke [18], and have been subject of three systematic reviews [17,19,20]. In short, these overviews have shown a benefit of light therapy. The Cochrane meta-analysis [19] which includes studies using light as an adjunctive treatment to antidepressants and to sleep deprivation or both, found a modest benefit of light treatment for non-seasonal depression in general, thus confirming a therapeutic benefit. An American Psychiatric Association work group concluded that efficacy appears equivalent to that of antidepressant drugs [17].

Martiny and others [21] investigated bright light compared with dim red light as adjuvant to 50 mg sertraline in a large sample (n = 90) of nonseasonal depression (mean age 45 years) and found response rates of 66,7% v. 40,7% and remission rates of 41,7% v. 14,8% for bright v. dim light, statistical significant differences favouring bright light treatment in clinical rated depression [21] scores and patient reported symptoms, and well being scales [22]. Goel and colleagues [23] gave 5 weeks morning bright light therapy (10,000 lux, 1 hour) to outpatients with chronic major depressive illness of ≥ 2 years who achieved remission rates of 50%; a control group given low-density negative air ionization showed only minor improvement.

The Committee on Chronotherapeutics [24] delegated by the International Society for Affective Disorders (SAD) concluded that light therapy hastens and potentiates the antidepressant response as an adjuvant to conventional antidepressants. Light therapy shows benefit even for patients with chronic depression of 2 years or more, outperforming their weak response to drugs. It should be noted, though, that all available reviews emphasize the need for further study because of the great diversity of short-duration study designs and relatively small sample sizes, all available overviews emphasized the need for further studies (e.g. [20,25]).

### BLT in geriatric nonseasonals

Most studies in elderly patients focussed on BLT as a therapeutic means to alleviate disruptive and cognitive symptoms of senile dementia. Recent reviews on the beneficial effects of BLT on sleep and behaviour note that the results of these studies are far from conclusive [26,27] Few empirical studies were performed in geriatric depression per se. A small crossover study (N = 10) in institutionalized patients with moderate-to-high Geriatric Depression Scale scores [28] tested morning bright (10,000 lux) versus versus dim light (300 lux), 30 minutes, 5 days, and obtained significant mood improvement under the active condition. However, these patients had no diagnosis of major depressive disorder. In the tropical climate of Taiwan, a trial of hospitalized patients with MDD (N = 30) [29] found alleviation of depressive symptoms after 5 days of morning light treatment (5000 lux, 50 minutes) in comparison with an untreated control group. However, the largest randomised controlled clinical trial in older patients (N = 81, 5 weeks) [30] found no significant benefit of bright light (10,000 lux, 1 hour; individualized according to pre-treatment phase typing into morning, midday, or evening application) over a 10-lux dim red control. Thus, results from studies on the effectiveness of BLT for geriatric depression are not conclusive.

### How light therapy may work

The neurobiological effect of light is mediated through the retina of the eye which is connected to the suprachiasmatic nuclei (SCN) situated just above the chiasmatic crossing of the optic nerve through the retinohypothalamic tract. The SCN is considered the major circadian pacemaker in mammals. Neural connections from the SCN are connected to the pineal gland and by this pathway the melatonin secretion of the pineal gland can be suppressed by light [31]. To date, the mood enhancing mechanism of bright light remains unknown.

### Wavelength

There are some indications that certain wavelengths of light are more effective than others in promoting a therapeutic response with the fewest side effects. This could be due to the ability of certain wavelengths of light to more effectively control circadian rhythms [32]. Recent studies have found that light in the blue spectrum (446–477 nm) outperforms other wavelengths in melatonin suppression, circadian phase shifting, and antidepressant effects [33,34].

### Ocular safety

Long-term follow-up of patients with cumulative exposure durations up to 1250 h demonstrated the ophthalmologic safety of light therapy, at least in patients without pre-existing ocular abnormalities [35].

### Several ways to address the placebo issue have been developed

The difficulty of creating a valid placebo condition is one of the main difficulties in light treatment trials. Eastman et al [36] and Goël et al [23] used a deactivated air ionization generator as a placebo control and checked that the daily behavioural commitment was equal to the one for light treatment and that the expectations of the patients were similar.

In SAD, Lewy et al [37] and Terman et al [38] used credible placebos by exposing the subjects to bright light at inappropriate times of the day. However, in nonseasonal depression, it appears impossible to predetermine an appropriate or inappropriate (placebo) time for light sessions as long as no single chronobiological phase alteration had been associated with depression. In the future it may well be possible to overcome this by selection of subgroups of chronobiologically homogenous patients or chronotypes. Only the Loving et al. [30] study phase-typed elderly nonseasonal depressed patients and individualized timing of light sessions according to clinical actigraphy criteria. However, the effect of light on melatonin phase appeared to be weak, suggesting low compliance or increased resistance to light in elderly patients.

### Antidepressant medication and bright light

In sum, bright light therapy might offer a safe, non-pharmacological alternative for elderly patients with a major depressive disorder. The few systematic reports of side-effects suggest it is safe with only a few counter indications. Light therapy may also provide a viable alternative for patients who refuse, resist or cannot tolerate medication. Finally, it may hasten and potentiate the antidepressant response when used as an adjuvant to conventional antidepressants. Therefore, the aims of the current study were to investigate whether bright light therapy is effective treatment in elderly outpatients suffering from a nonseasonal depressive disorder, when being used as stand-alone or as an adjuvans to antidepressive therapy.

## Methods and design

### General objectives

The purpose of this study is to investigate the following hypotheses:

1. Treatment with bright light improves mood, sleep, concentration and self-sufficiency of elderly depressed subjects. This clinical improvement is accompanied by decreases in cortisol and increase in melatonin concentrations.

2. The eventual beneficial effect of bright light treatment can be predicted by the presence of sleep-wake rhythm disturbances as found using muscle activity registration, and by cortisol and melatonin concentrations in saliva and urine over the day and the night.

### Design

**Figure 1:**
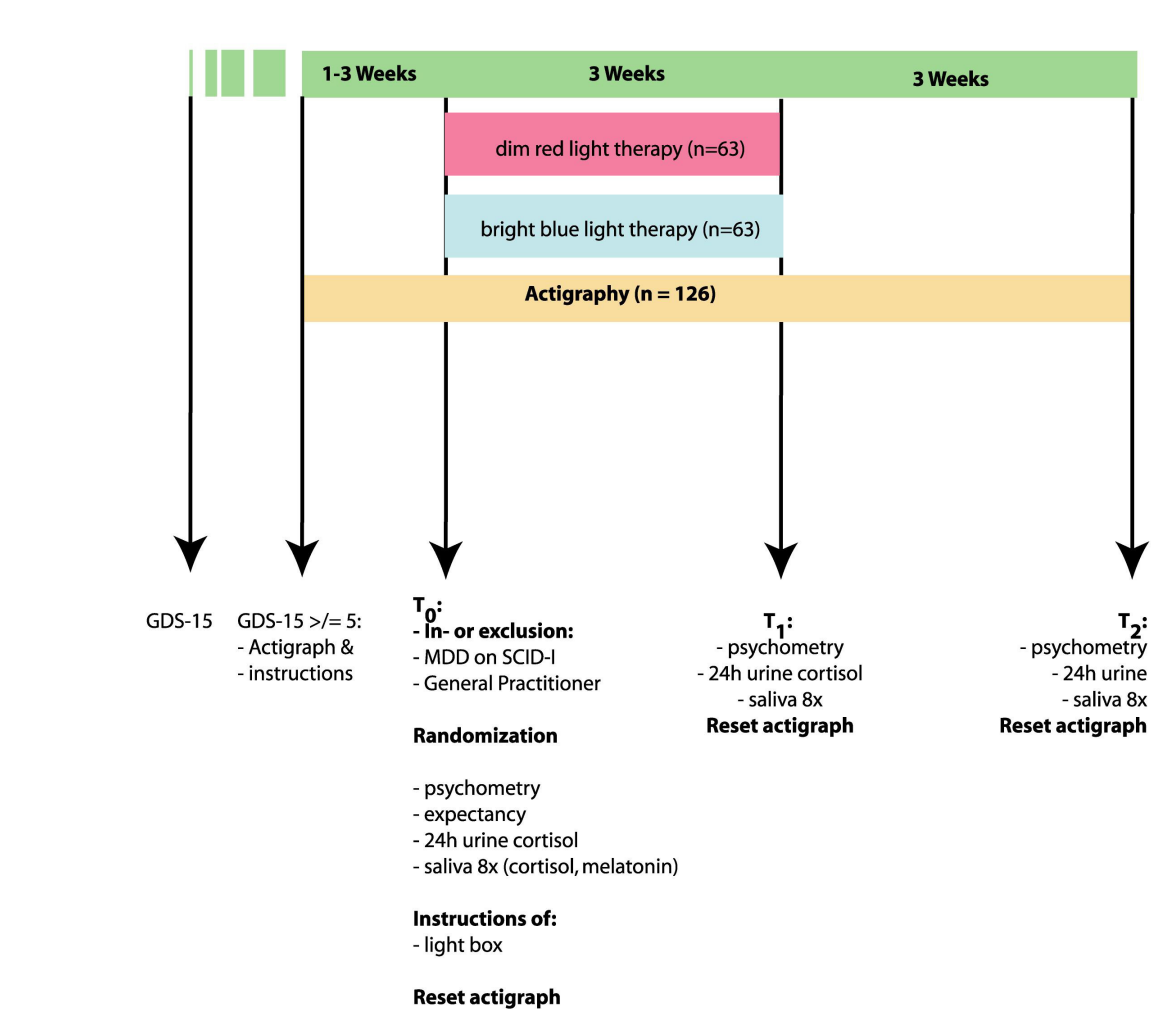
Scheme-diagram of the trial protocol.

A scheme-diagram of the trial protocol is presented in figure 1.

### Participants

Volunteers are recruited in the Amsterdam region using four strategies: (1) referrals from several outpatient psychiatry secondary care settings specialized in elderly care in the Amsterdam region, (2) referrals from general practitioners, (3) advertisements in local newspapers, folders from 25 apothecaries and waiting rooms of general practitioners, (4) an active case-finding procedure by screening elderly populations from 16 General Practitioners Offices using the 15-item version of the Geriatric Depression Scale (GDS-15, [39]). Subjects with a GDS-15 score of five or more or with depressive symptoms are interviewed by telephone and/or a live interview to establish whether they fulfil the eligibility criteria.

### Inclusion and exclusion criteria

The inclusion criteria are 60 years or more of age, a primary diagnosis of Major Depressive Disorder according to DSM-IV criteria, as assessed with the Structured Clinical Interview [40] by a trained research physician (RL) or a specially trained research psychologist.

Candidates are not admitted to the study if any of the following criteria were present: (1) insufficient fluency in Dutch, (2) living outside the Amsterdam region, insufficient address for follow-up, or unwillingness to return for follow-up, (3) light treatment in the past, (4) history of light-indicated migraine or epilepsy, (5) presence of physical illnesses that require urgent specialized treatment (e.g. untreated diabetes, malignancies, chronic infections, thyroid problems, eye diseases (glaucoma, cataract) for which surgery is scheduled in near future), (6) the presence of other major axis-I disorders like bipolar disorder, dementia, delirium, all psychotic disorders, PTSD, a recent history of a suicide attempt, the presence of the DSM-IV Seasonal Pattern specifier, (7) diseases that could interfere with HPA-axis functioning, or (8) diseases or medication that could elevate the risk of light-therapy associated side effects (e.g. progressive eye diseases, senile macula degeneration, Lupus, tricyclic antidepressants, tetracyclic antibiotics), (9) current drug or alcohol abuse less than 3 months before the study, (10) any drugs (e.g. corticosteroids, cyclosporin A) known to interfere with endocrine function. Also excluded were all subjects who were taking (11) SSRIs or SNRIs shorter than 2 months.

### Interventions

We chose to use dim red light as a placebo, reasoning that because the red part of the spectrum is biologically relatively inactive, there would be no substantial effect. Another advantage of red light as placebo is that it induces very strong positive expectations quite comparable to those of bright white light [41,30].

Patients receive either early morning bright blue light or early morning dim red light. Portable light boxes are used with a fixed (non-tuneable) light-intensity (Philips Bright Light Energy^® ^boxes type HF 3304). Mistblue filters (Leevellen Lee 061) are built in to arrange the active bright-light condition. Blood-red filters (Leevellen Lee 789) are built in for the placebo dim-red light condition. On the outside of the boxes no difference is observable between the two conditions. Emitted light spectra and intensities are quantified using a lux meter at 40 cm from two light boxes. Bright light consists of approximately 10.000 lux. Dim red light consists of less than 50 lux. Environmental light in the subject's home at the designated sitting place of light therapy is measured at the start of the light therapy using a lux-meter.

We offer a 3 week treatment period of 1 hour of light therapy each day. At installation of the light devices, patients choose a 3-week-lasting fixed starting time anchored within 1 hour from their habitual wake-up time. Clock-programmable power supply of two light-boxes is set to switch 'on' at that time-point every day during the 3-week period and 'off' after one hour.

### Compliance

Compliance to light therapy is retrospectively measured by lux-meters that were built in the wrist-worn actimeters patients wear during the entire protocol. Subjects are instructed by a protocol-blinded instructor to sit at their table, with the light boxes put at a distance of approximately 40 cm from the eyes. During therapy, subjects sit at the table to have breakfast or do some reading (e.g. news paper). Participants will be asked to note their compliance in their trial-diaries.

### Sample size

Martiny et al. [21] found a moderate effect size (ES>0,50 maximum 0,66) for bright light treated group in comparison with dim light treated group on both self-assessment and observer rating scales in patients with a high prevalence of melancholic depression. Based on the literature on the expected response rate, and using conventional values for α (0.05) and β (0.80), and two-tailed tests with equal groups, the sample size aimed for in the current study is 63 patients per arm [42].

### Randomization

Randomisation was prepared by an independent researcher (BU) using a computer generated table, blocking in subsets of 10, transcribed to closed envelope messages. Stratification is applied for use antidepressant medication (SSRIs and SRNIs were allowed). After inclusion the researcher sends an email with name, contact information and condition i.e. AD+ or AD-) to one of our two specially trained protocol-blinded instructors. As a check the instructor writes the allocated condition onto a paper which is kept in a special envelope in the same box with the closed envelopes.

### Blinding

The study is presented to the patients to study whether light therapy would be a beneficial treatment modality for major depressive disorder. The blue light and red light conditions are presented to study whether a specific part of the light spectrum would be more effective than another.

Light treatment is blinded to the investigators as the lamps are delivered at the patient's homes by a specially trained protocol blinded instructor, so the instructors are led to believe that the blue and red light conditions are to study which part of the spectrum worked better than the other.

The patients are asked not to refer any details of their condition to the investigators as this would harm the study. When patients however do reveal their condition, the researcher will be replaced immediately by another researcher to perform psychometrical interviewing and ratings.

To evaluate the blinding procedure, and in order to assess participants' general expectations for light therapy and their specific expectations in respect to their allocated light boxes, we measure expectations before the light box was installed using a four item expectations questionnaire. Briefly, participants rate, on a 7-points scale (1 = feeling much better, 2 = feeling definitely better, 3 = feeling slightly better, 4 = no change, 5 = feeling slightly worse, 6 = feeling definitely worse, 7 = feeling much worse), how they thought light therapy would improve their symptoms. We use four questions to be rated: (1) To what extent do you expect your problems will improve without treatment? (2) To what extent do you expect your problems will improve with light treatment? (3) To what extent do you expect your problems will improve with the blue light treatment? (4) To what extent do you expect your problems will improve with the red light treatment?

### Statistical analysis

Data will be analyzed using SPSS 16.0. Analyses are performed using intention-to-treat principles. Therefore, drop-outs are distinguished into 'drop-outs before T1 assessment' and 'drop-outs after T1 assessment'. When only a randomisation assessment (T0) is available, subjects are considered lost to follow-up. For 'drop-outs after T1 assessment' the principle of LOCF (last observation carried forward) will be used. For sensitivity analysis, the more conservative LOCF variant will be calculated where T0-data will be carried forward, and a completers analysis will be performed.

Baseline characteristics across the treatment groups are compared using analysis of variance (ANOVA) and χ^2^-statistics.

As the primary analysis efficacy of treatment is tested by performing a repeated measures analysis of variance (RMANOVA) on HADRS-17 scores at T0, T1 and T2. To control for possible difference in baseline severity scores baseline values for HADRS-17 are used as covariates. Repeated contrasts are applied to compare effects in the treatment period with effects after the treatment period. As secondary analyses efficacy of treatment is tested by performing RMANOVA on the other depression symptom scales (SIGH-SAD scores, HAM-D6, MADRS-scores). As tertiary analyses depression outcome measures are dichotomised to responders and non-responders, where response is defined as a decrease of ≥ 50% on the HADRS-17, HAM-D6, SIGH-SAD and MADRS scales. Responder rates will also be presented as odd's ratios and NNT's.

### Approval

The current study is executed in accordance with the principles laid down in the Helsinki Declaration [43]. Participation in the study is voluntary, and written informed consent is obtained. The patients are explicitly informed of the fact that they can withdraw their consent to participate at any time, without specification of reasons and with no negative consequences with regard to their future medical treatment. Patients who wish to withdraw from the study will receive care as usual. Approvals were obtained by the Dutch authorities and the medical ethical committee of GGZ Nederland.

### Primary Outcome measure

Depression severity is assessed by a trained research physician and qualified research psychologists (MN, RdV) using the Montgomery-Åsberg Depression Rating Scale (MADRS) [44], the combined 21-item Hamilton and 8-item atypical symptom scales of the Structured Interview Guide for the Hamilton Depression Rating Scale – Seasonal Affective Disorder Version (SIGH-SAD) [45], which although originally designed for SAD studies is equally applicable for assessment of nonseasonal depression with atypical features (as in the present study). The Hamilton and atypical symptom subscales of the SIGH-SAD can be analyzed separately or in combination [46]. The primary outcome measure is depression ratings using the HADRS-17 scale. Response is defined as a 50% reduction in depressive symptoms, on the HADRS-17 scale at T1 and T2. Specially trained clinical research staff conducts the interview.

### Secondary Outcome measures

Secondary outcome measures are clinical response (i.e. 50% reduction in depressive symptoms) on the 29-items SIGH-SAD, the Atypical-8 subset from the SIGH-SAD, the HAMD-6 depression core features subscore, and the MADRS. Specially trained clinical research staff conducted the interviews.

### Adverse effects

At each visit and 4 additional times, subjects are systematically asked about possible side effects by the blind raters: early morning awakening, headache, agitation, drowsiness, irritability, tight muscles, nervousness, anxiety, tremor, dizziness, fatigue nausea, diarrhoea, dry mouth, anorexia, dyspepsia, constipation, excessive sweating, rash, asthenia, viral infection, upper respiratory infection, flulike syndrome, nasal congestion and hot flushes [47]. Each item is rated as absent, mild, moderate or severe. The subjects were instructed to report any side effects that could have jeopardized the blindness of the raters (such as eye strain, a side effect characteristic of bright light) to a nonblind research assistant who simply tabulated these side effects.

### Endocrine measures

1. Activity of the HPA-axis was assessed by free cortisol assessment using 8 saliva samples per respondent per time. As a measure of a natural "stress" response of the HPA-axis, the morning cortisol awakening response (CAR) is assessed by taking saliva samples at T0, T1 and T2 (at get-up time plus 30 minutes, plus 60 minutes, plus 90 minutes, plus 120 minutes), and the cortisol evening curve (bedtime minus 4 hours, minus 3 hours, minus 2 hours, minus 1 hour). Saliva samples are collected using cotton swaps (Salivettes, Sarstedt, Germany). Subjects are instructed to be seated during the last 15 minutes before saliva collection. During the entire sampling period, subjects can watch TV or perform leisure activities. Eating and drinking (with the exception of coffee, tea, chocolate, and bananas), and smoking will be permitted. Saliva containers are kept in the patient's refrigerators and are to be collected by the psychometrist the next day after psychometry, which are delivered to the clinical labwhere it is spinned down and stored at -85°C. Cortisol will be assayed on an Elecsys using a standard cortisol kit (Roche). Saliva samples are frozen in order to do all measurements at once.

2. To determine a patients circadian phase position the Dim Light Melatonin Onset (DLMO; the time point when melatonin secretion rises over a predefined threshold in the evening, under experimental dim light conditions) will be calculated after measuring the melatonin evening curve using measurements in saliva samples (bedtime minus 4 hours, minus 3 hours, minus 2 hours, minus 1 hour) at T0, T1 and T2.

3. Total cortisol excretion at T0, T1 and T2 will be determined using 24-hour urine collections. Collections are carried in the participants' own homes, following procedures provided through detailed written and verbal instructions. Urine is collected for 24 hours after the first voided urine following awakening, and included the first voided urine on the following day. Three liter polyethylene collection bottles were used. The completeness of data collection is ascertained by measurements of the 24-hr urine volume and creatinine excretion. Only participants demonstrating good compliance, using this quality control step, will be included in the analyses. The urinary free cortisol (UFC) will be determined by radioimmunoassay using a commercially available (Coat A Coat, Diagnostic Product Corporation (DPC), Los Angeles USA) antibody specific for cortisol and I-125-labeled antigen. Urinary measurements are performed by the laboratory at arrival.

### Actigraphy

Before, during treatment and three weeks after treatment circadian rest-activity will be measured using actography. The small (57 × 46 × 22 mm) and light-weight (70 g) wrist-worn actigraph, which senses movement-induced accelerations, as described elsewhere [48].

Estimates of sleep parameters are obtained using the validated Sleepwatch Analysis Software 201 (Cambridge Neurotechnology Ltd., Cambridge, UK). The software automatically calculates Sleepstart and Sleepend, which are limited to occur at any time between the habitual Bed Time and Get Up Time as read from the sleep logs and entered into the software; Assumed sleep (the difference between Sleep End and Sleep Start), Actual Sleep time (amount of sleep determined by the algorithm and equivalent to assumed sleep minus wake time. We defined Sleep Efficiency bas the percentage of actual sleep time between sleep onset and final awakening, excluding sleep onset latency. Mean Activity scores are average activity scores in those epochs where scores of greater than zero are recorded during the assumed sleep period.

Circadian rest activity pattern is quantified using several actimetric variables. The Interdaily Stability (IS) gives an indication of of the stability of the 24-hour rest activity pattern over days. The intradaily variability (IV) gives an indication of the fragmentation of the rhythm, by quantifying the number and strength of transitions between periods of rest and activity. L5 quantifies the activity level during the core sleep period. M10 describes the daytime activity level. AMP is an absolute amplitude measure and calculated as the difference between M10 and L5. The relative amplitude (RA) is calculated by dividing AMP by the sum of L5 and M10 [48,49].

### Sleep analysis measures

Subjective sleep quality is scored using the Pittsburgh Sleep Quality Inventory (PSQI) at T0, T1 and T2 [50]. The PSQI is a validated self-rated questionnaire which assesses sleep quality and disturbances. Nineteen individual items generate seven "component" scores: subjective sleep quality, sleep latency, sleep duration, habitual sleep efficiency, sleep disturbances, use of sleeping medication, and daytime dysfunction [50]. PSQI-scores can be dichotomized as follows: PSQI >/= 5 indicate poor sleep, if PSQI <5 indicates good sleep.

### Circadian rhythm measures

An abbreviated version of the Social Rhythm Metric SRM-17 [51], the SRM-5 [52] was translated back and forth as there was no validated Dutch translation available. It will be used to quantify the daily rhythms of life or daily lifestyle regularity. These items are: (1) Get out of bed, (2) First contact with another person, (3) Start work, housework or volunteer activities, (4) Have dinner, and (5) Go to (bed) [52]. From this diary a weekly SRM-score can be measured, which is a validated measure of lifestyle regularity, yielding the scores of one to three weeks before treatment, the three treatment weeks and scores from the three weeks after treatment.

### Neuropsychological tests

At T0, T1 and T2 participants will do neuropsychological tests addressing working memory, using the digit-span forward and the digit span backward, learning and memory using the Rivermead stories immediate and delayed recall, and verbal fluency using animals, insects and occupations.

### Questionnaires

Details about the social support is measured using Social Support Questionnaire (SSQ) [53], at T0, T1 and T2; The SSQ has 41 items on social support interactions, 41 items on social support discrepancies and 7 items on negative interactions. It focuses on seven domains of social support: emotional Support (four items, refer to the emotional support of everyday life, like showing affection), social support by problems (eight items, contains items like cheering and backing up), informative support (four items, refers to information about one's behaviour like making the subject understand why something they did was wrong or making clear what is expected of him-her), instrumental Support (seven items, refers to financial and material support, like helping with practical matters, e.g. shopping or lending small things or Money), Social Companionship scale (five items, are inviting the subject for dinner or a party and calling him/her up for a chat), Esteem Support (six items, refer to being valuated by others, with items like asking advice or showing confidence).

In order to measure self-sufficiency we chose to use the MOS-short form General Health Survey (SF-20; abbreviated form of the RAND-36) [54]. De SF-20, like RAND-36, is a multi-dimensional instrument to measure general health. The SF-20 is shorter than RAND-36 and aims on different aspects of health, e.g. the time of certain health related impairments. In Groningen SF-20 is widely used in large studies among elderly people. It contains scales for physical, social functioning, role impairments due to physical or emotional problems, mental health, energy, pain and general perception of health [54].

To additionally measure both the ADL and instrumental ADL disability with respect to the aid and services provided by professional home help and district nursing agencies we will use the Groningen Activiteiten Restriction Scale (GARS; Kempen et al 1993).

The general self-efficacy was measured with the Dutch version of the General Self-Efficacy Scale (GSES) the Algemene Competentie Schaal (ALCOS) [55]. The ALCOS-12 as well as the subscales have earlier been shown to be moderately reliable and valid instrument to measure (aspects of) generalized expectations of self-efficacy.

A Dutch version of the Philadelphia Geriatric Center Morale Scale [56] is used to measure well-being and life-satisfaction at T0, T1 and T2.

### Neuroimaging

A subset of consecutively included participants is invited for 1,5 Tesla MR-scanning at T0 and after therapy at T1. Scan protocols consist of structural MPRAGE/T1- and T2 weighted magnetic resonance scans of the brain, functional-MRI scans using encoding, facial recognition task and working memory tasks (N-back). In addition, adrenal volumetry is performed using abdominal MPRAGE of adrenals.

## Abbreviations

AD: Antidepressant; BLT: Bright light therapy; CAR: Cortisol awakening response; DLMO: Dim Light Melatonin Onset; DSM-IV: Diagnostic and Statistical Manual of Mental Disorders 4th Edition: GDS-15: Geriatric Depression Scale 15item version; HAMD-6: 6-core-item subscore from HDRS-17; HDRS-17: Hamilton Depression Rating Scale 17 item version; HPA-axis: Hypothalamus-pituitary adrenocortical axis; MADRS: Montgomery-Åsberg Depression Rating Scale; MDD: Major depressive disorder; MPRAGE: Magnetization Prepared RApid Gradient Echo; SAD: Seasonal affective disorder; SCID-I: Structured Clinical Interview for DSM-IV; SCN: Suprachiasmatic nucleus, SIGHSAD: Structured Interview Guide for the Hamilton Depression Rating Scale – Seasonal Affective Disorder Version; UFC: Urinary Free Cortisol.

## Competing interests

The authors declare that they have no competing interests.

## Authors' contributions

RL is the principal investigator, participated in the study design, patient recruitment and trial coordination, drafted the manuscript, MMAN is the postdoc, participated in the study design, patient recruitment and participated in the trial coordination, DJV participated in the neuroimaging design, BU is a neurologist-epidemiologist and participated in the study design, set up the randomisation system, EJWS is psychophysiologist, conceived of the study, and participated in the study design, conceived the fabrication of the trial conditions, and supervisor of actometry, JHS is the trial methodologist and chief research of the academic department of psychiatry, participated in recruitment strategies, in methodology and interpretation issues, WJGH is the study director, conceived of the study, obtained funding, participated in design and coordination and helped on the manuscript. All authors read and approved the final manuscript.
